# Designing of novel chimeric PvpA-pMGA protein of *Mycoplasma gallisepticum*, applicable for indirect ELISA

**DOI:** 10.1186/s43141-022-00434-0

**Published:** 2022-11-29

**Authors:** Farzaneh pourkarimi Fatideh, Majid Esmaelizad, Mohammad Kargar, Majid Tebianian, Farshid Kafilzadeh

**Affiliations:** 1 Department of Microbiology, Jahrom Branch, Islamic Azad University, Jahrom, Iran; 2 Research and Development Department, Razi Vaccine and Serum Research Institute, Agricultural Research, Education and Extension Organization (AREEO), Karaj, Iran; 3 Department of Immunology, Razi Vaccine and Serum Research Institute, Agricultural Research, Education and Extension Organization (AREEO), Karaj, Iran

**Keywords:** *Mycoplasma gallisepticum*, Chimeric protein, Immunogenicity, PvpA-pMGA, Recombinant protein, ELISA

## Abstract

**Background:**

*Mycoplasma gallisepticum* is the primary agent of chronic respiratory disease in chickens creating important economic losses in poultry industry. pMGA and pvpA genes encode major surface proteins in *M. gallisepticum* containing pathogenic, antigenic, and immune evasion characteristics. The objective of the present study was to design, express, and purify the recombinant chimeric PvpA-pMGA protein from *M.gallisepticum* for using in serological diagnostic test.

**Methods:**

Antigenic regions of PvpA and pMGA proteins were predicted for designing chimeric pvpA-pMGA gene construct. The codon optimized sequence was cloned into the expression vector pET32a+ and transformed into the *Escherichia coli* strain BL21 (DE3). The pET32a-PvpA-pMGA recombinant plasmid was expressed and confirmed by SDS-PAGE and immunoblotting. PvpA-pMGA recombinant protein (20μg and 50μg), ts-11 vaccine strain, and S6 strain that formulated by montanide adjuvant and two control groups (PBS and adjuvant) were injected subcutaneously to six groups of chickens.

**Results:**

High yield of protein was purified amount 138 mg/L by affinity batch formation method. Indirect ELISA showed the levels of antibodies in rPvpA-pMGA was significantly higher than ts-11 and S6 groups (*p*<0.05). The results indicated that antigen-specific response was successfully elicited by the rpMGA-PvpA in chickens. The result of the ELISA with sera collected from ts-11 and S6 groups showed that indirect PvpA-pMGA-ELISA is appropriate candidate for detection of specific antibodies against *M. gallisepticum* with 100% sensitivity and specificity.

**Conclusions:**

The rPvpA-pMGA is a highly candidate immunogenic protein which induced high amount of humoral immune response. Novel rPvpA-pMGA protein could be useful for evaluation of antibody level in vaccinated poultry flocks.

## Highlights

• This is first study to present chimeric protein rPvpA-pMGA as a candidate antigen in ELISA.

• High-yield 138 mg/L purification of recombinant protein.

• rPvpA-pMGA is a reliable immunogenic protein which induced high amount of humoral immune response.

• rPvpA-pMGA showed high performance for evaluation of antibody level in vaccinated poultry flocks.

## Background

*Mycoplasasma gallisepticum* is a super respiratory pathogen of chickens and turkeys belongs to the category *Mollicutes,* the cell-wall-less eubacteria [47], and leading to severe economic losses in poultry trade [32, 51]. *C*oughing, sneezing, nasal and ocular discharge, reduction in feed intake, and expansion in mortality are clinical indications of mycoplasmosis [11, 40]. *Mycoplasma gallisepticum* infection leads to chronic respiratory disorder, infectious sinusitis, diminished weight gain, decline in production and egg quality, and increase in production costs for poultry producers [17, 30]. Past reports showed that *Mycoplasma gallisepticum* infection can occur in ducks [8], geese [9], pheasants, partridges, peafowl, pigeons, sparrows, and finches [23, 31, 33]. Other studies revealed that the backyard poultry flocks could act as reservoir or amplifier for poultry respiratory infections serving as a nonstop wellspring of infection for industrial chickens [22, 47].

Numerous studies, focused on killed vaccines, live-attenuated vaccines, bacterin-based, or recombinant proteins [46, 55] to control *Mycoplasma gallisepticum* contamination. Colonization of *Mycoplasma gallisepticum* in the host respiratory tract; the presence of other respiratory pathogens, phase, or antigenic variation that lead to immune escape from the host immune system; and transmission of contamination in the flocks and immune status of the host are challenges that prevent the triumph of vaccines to forestall *Mycoplasma gallisepticum* infection [29]. Control of *Mycoplasma gallisepticum* has mainly been based on the elimination of the organism from flocks by occasional serological monitoring, following culture and PCR [33]. In some countries, where complete annihilation is troublesome, vaccination with live vaccines is utilized as an elective control system [28, 55]. Between serological tests, the rapid plate agglutination (RPA) test is extremely straightforward and delicate. For a more explicit and sensitive diagnosis of *Mycoplasma gallisepticum* enzyme-linked immunosorbent assay (ELISA) can be used [1, 30]. A multi-gene family contains 30 to 70 genes in mycoplasmas encoding the VlhA or pMGA immunodominant lipoproteins and hemagglutinins that undergo phase-variable expression [3, 4, 35]. The pMGA and PvpA proteins are phase variables and therefore contribute to the changelessness of *Mycoplasma gallisepticum* in the respiratory tract [34, 56]. The role of the pMGA protein, a 67-kDa putative hemagglutinin of *Mycoplasma gallisepticum*, in generating antigenic variation has been demonstrated with both phase variation during the acute phases of sickness and antigenic switching during the chronic stages in tainted chickens [20]. Nine individuals from the pMGA gene family in *Mycoplasma gallisepticum* S6 strain have been sequenced (pMGA_1.1_-pMGA_1.9_). The pMGA_1.1_ and pMGA_1.2_ genes have a high level of sequence identity (>95%), whereas all the other pMGA genes exhibit much lower overall identity [21, 41].

pMGA and cytadhesins MGC_1_, MGC_2_, and MGC_3_ are associated with the adhesion capacity of *Mycoplasma gallisepticum* [24, 45]. PvpA is a putative hemagglutinin and phase-variable protein recognized by the chicken immune system that undergoes phase-variable expression and shows size variation among strains of *Mycoplasma gallisepticum* [10, 29, 58]. Size variation of the *pvpA* gene was seen in strains of *Mycoplasma gallisepticum* as a result of deletions occurring in the segment encoding the proline-rich C-terminal region of the protein [10]. PvpA may play a role in the attachment of *Mycoplasma gallisepticum* to the chicken trachea [10]. The roles of PvpA, pMGA [38], MGC_2_ [24], GapA [45], and CrmA [25, 44] proteins in the cytoadherence and virulence of *M. gallisepticum* have been identified [39, 58].

Several mAbs that perceive epitopes in different locales of the pMGA_1.1_ or pMGA_1.2_ proteins can forestall hemagglutination of different *Mycoplasma gallisepticum* strains, indirectly demonstrating that pMGA is engaged with the limiting of *M.gallisepticum* to erythrocytes [7, 38]. Since PvpA is available to the host immune response, this surface-exposed protein could be a potential diagnostic antigen of the decision in *Mycoplasma gallisepticum* contamination [10] and it’s species-specific and immunogenic properties have significantly been demonstrated [58]. Jan et al. in 2001 analyzed surface proteins P67 (known as pMGA) and P52 of *Mycoplasma gallisepticum*, and the results indicated that P67 is a genuine membrane-related lipoprotein pMGA_1.2_ and after-effects of immunoblotting corroborated surface protein P67 (pMGA) was specific to *Mycoplasma gallisepticum* [26]. Therefore, in this present study, we attempt to design a chimeric PvpA-pMGA_1.2_ protein to develop a recombinant antigen applicable to indirect ELISA.

## Methods

### Bioinformatics studies

Two pMGA and pvpA major surface proteins with high antigenic index and specific for *M. gallisepticum were selected for DNA construct design.* A total of eighteen complete genomes of *Mycoplasma gallisepticum* available in GenBank (https://www.ncbi.nlm.nih.gov/GenBank) till January 2020 were collected. The complete coding sequences of two pMGA and PvpA gene were selected from complete genomes. Multiple alignments of protein sequences were performed by MegAlign 5.00 DNASTAR Inc., and software and dominant sequences with maximum coverage were selected. Antigenic regions of two proteins were predicted by IEDB (Immune epitope database and analysis resource) tools. Antigenic regions were assembled together and hydrophobic linkers (ggggs) were added between fragments of PvpA and pMGA. Then, for efficient purification of PvpA-pMGA protein, a 6X-His tag sequence was considered to carboxyl terminus of the designed protein. Physical and chemical parameters include the molecular weight, theoretical pI, amino acid composition, estimated half-life, instability index; aliphatic index, and grand average of hydropathicity (GRAVY) of chimeric protein were analyzed.

The nucleotide coding sequence of the chimeric protein was optimized for expression in *E. coli* by IDT codon optimization tool. Restriction sites were improved based on recognition sites of pET32a+ expression vector. Two EcoRI and XhoI restriction sites were considered in 5′ and 3′ ends of DNA for subcloning into the vector. The DNA with 876 bp length was synthesized in ShineGene Molecular Biotech, Inc., China.

### Expression of PvpA-pMGA recombinant protein

The recombinant plasmid of pET32a-PvpA-pMGA was transformed into the *E. coli* strain BL21 (DE3) as a host strain, on LB Agar containing 50μg/ml ampicillin by the heat shock method [48]. Then, a matrix plate was subcultured, and one single colony was cultured in 5-ml Luria-Bertani medium containing 50μg/ml ampicillin at 37°C overnight. This suspension was inoculated into a 50-ml 2YT broth medium containing ampicillin. Expression of the recombinant PvpA-pMGA protein was induced by the addition of 0.1mM isopropyl beta-d-thiogalactopyranoside (IPTG) to the culture when the optical density reached to 0.8 at wave length A_600_. Optimal conditions for the expression of recombinant protein were evaluated in different incubation times (0 to 16 hours), temperatures (22°C and 37°C), and various concentrations (0.1 to 0.5 mM) of IPTG.

The 1-ml samples which were collected from the bacterial culture before and after were harvested by centrifugation at 5000 g for 5 min at 4°C. The pellets were suspended in 1XPBS (137 mMNaCl, 2 mMKH_2_PO_4_, 10 mM Na_2_HPO_4_, 2.7 mM KCL, PH 7.4). Then boiled for 5 mins after the addition of sample buffer (5X solution of 250 mM Tris-HCL, pH 6.8, 10% SDS, 30% (v/v) glycerol, 10 mM DTT, 0.05% (w/v) bromophenol blue, 2-mercaptoethanol) and the presence of recombinant protein were analyzed by 10% SDS-PAGE.

### Purification of pMGA-PvpA recombinant protein

Induced cells were resuspended in 1XPBS buffer. Sonication was performed for 1 min five times on ice with 1 min intervals to lyse the cells. Then, to prevent the activity of protease, 1mM of phenyl methyl sulfonyl fluoride (PMSF) was added. Post-sonication cell lysate was centrifuged at 15,000 g for 15 min at 4°C. After that, the supernatant and pellet were analyzed by SDS-PAGE to check the expression of recombinant protein.

The recombinant PvpA-pMGA chimeric protein was resuspended from the inclusion body pellets by the addition of 7M urea and incubated in a shaking incubator at 37°C for 16 h. Then, centrifugation was performed at 15,000g for 15 min at 4°C. The supernatant was used to affinity batch formation method by nickel resin. The affinity chromatography was done by adding 200μl Nickel resin (Thermo Scientific) into the supernatant. Then, it was shaken for 1 h at room temperature and centrifuged at 3000g for 5 min at 4°C. The supernatant was removed, and the pellet was washed thrice with 500-μl washing buffer (20 mM Tris, 500 mM NaCl, 50 mM imidazole, pH 8.0) and centrifuged at 3000g for 5 min at 4°C. The His-tagged protein was eluted with 200μl elution buffer (20 mM Tris, 500 mM NaCl, 200 mM imidazole, pH 8.0) and shaken for 15 min. The recombinant protein was eluted five times by centrifugation at 3000g for 5 min at 4° C. The quantity of the purified recombinant PvpA-pMGA was determined by Bradford protein assay [12].

### Immunoblotting

Chimeric protein PvpA-pMGA was electrophoresed into the 10% SDS-PAGE. Protein was transferred from SDS-PAGE to the nitrocellulose membrane in transfer buffer (25 mM Tris, 192 mM glycine, 20% v/v methanol, pH 8.3) for 2 h at 100 v. The membranes were blocked with shaking in 1% BSA in PBST (phosphate-buffered saline, pH 7.4;0.1% Tween 20) for 1 h at room temperature. After washing with PBST, the membrane was shaken in1:5000 dilution of HPR conjugated anti-His Tag antibody (Abcam, USA) for 1 h at room temperature. The chromogenic reaction was developed by 4-chloro-1-naphthol (Sigma, Germany).

### Chicken immunization and detection of antibody

One-day-old SPF chickens (*n*=60) were obtained from the SPF-egg production unit of the Razi Vaccine and Serum Research Institute. After 4 weeks, chickens were divided randomly to six groups (ten chickens each): 50μg rPvpA-pMGA (rPro1), 20μg rPvpA-pMGA (rPro2), ts-11 vaccine strain, and S6 strain as a positive control, adjuvant (Montanide) and PBS as a negative control. The purified recombinant PvpA-pMGA_1.2_ protein (20μg and 50μg) was emulsified with Montanide^TM^ ISA 71 VG at a 3:7 ratio (antigens to adjuvant) for two groups rPro2 and rPro1, respectively. Chickens in the vaccine group were immunized with 0.5 ml ts-11 vaccine strain of avian *Mycoplasma gallisepticum*. Chickens in the PBS group were injected with 0.5-ml phosphate buffer (0.01 M, pH 7.2). The chickens in the adjuvant group were injected with 0.5-ml adjuvant plus PBS. The root of the injection was considered subcutaneous (SC) with a dosage of 0.5 ml volume at the back of the neck for all groups. The chickens in each of the above groups were immunized three times with 2 weeks of intervals. The levels of specific antibodies were determined by indirect enzyme-linked immunosorbent assay (ELISA).

After immunization, blood samples were collected from the brachial wing of immunized chickens before and 14, 28, and 42 days after injection and centrifuged to obtain serum. The optimal serum dilutions and recombinant antigen concentrations were determined by a checkerboard titration. The polystyrene 96-well microtiter plate (Nunc) was coated overnight at 4°C with 100 ng of recombinant PvpA-pMGA protein in 100 μl/well carbonate-bicarbonate buffer (pH 9.6). Unoccupied protein binding sites were blocked by adding 300 μl of blocking buffer (5% skim milk to PBS) to each well and the plate was incubated for 1 h at 37°C. After incubation, wells were washed three times with PBS-T(PBS plus 0.05 % Tween20). Then, the plate was incubated 1 h at 37°C with 100 μl of 1/50 diluted sera from immunized chickens. After additional washing steps with PBS-T, the goat anti-chicken IgG (SIGMA) in 1/6000 dilution was added to each well and incubated for 1 h at 37°C. After thrice washing steps with PBS-T, the color reaction was performed by adding tetra methyl benzidine (TMB) substrate solution and incubated for 10 min at room temperature in darkness. Finally, the reaction was stopped with a stop solution (2M H_2_SO_4_), and the absorbance was read at 450 nm with the ELISA plate reader (Bio Tek, Elx 800).

### Statistical analysis

The statistical analysis was performed using GraphPad Prism Version 9.2.0 (GraphPad Software Inc., San Diego, CA, USA). Comparisons between the chicken groups for antibody levels were performed by one-way ANOVA, followed by Sidak’s test. *P* values *<*0.05 were considered statistically significant.

## Results

### Bioinformatic results

**Fig. 1:**
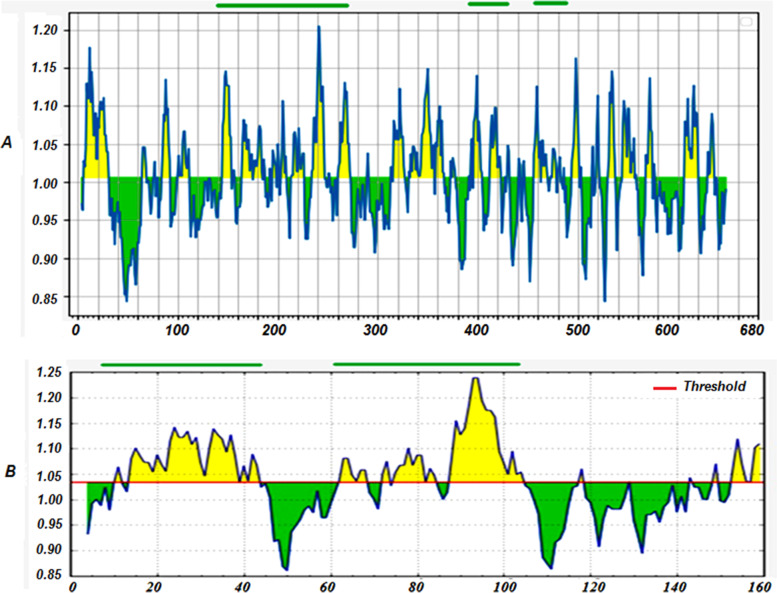
Antibody epitopes predicted by Kolaskar and Tongaonkar antigenicity method. **A** Three antigenic regions 144 to 274, 394 to 420, and 454 to 480 were considered in the pMGA protein. **B** Two antigenic regions 13 to 45 and 62 to 104 were chosen in Pvpa protein

**Fig. 2:**
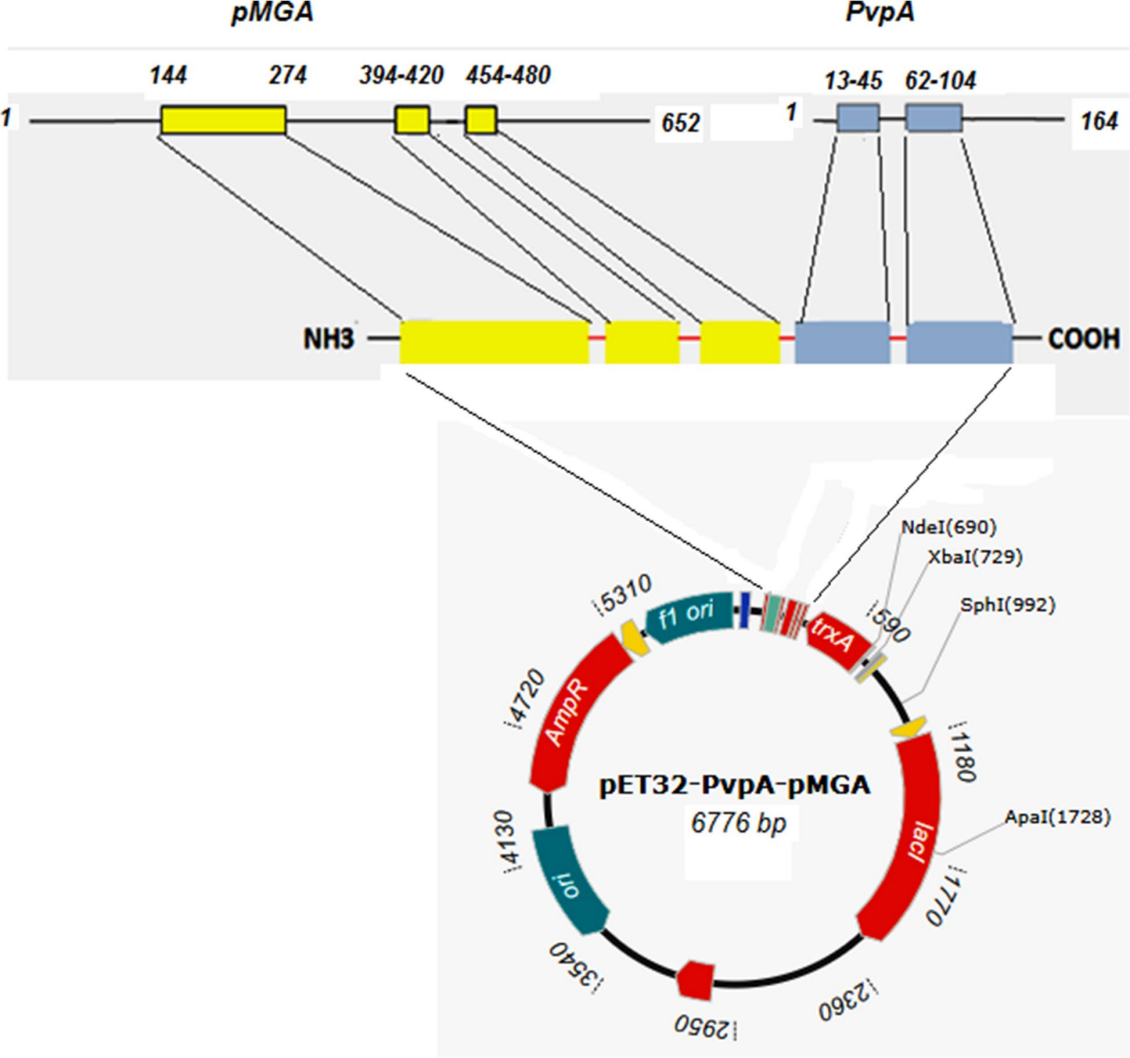
Schematic diagram of the designed chimeric PvpA-pMGA protein and expression vector. Five amino acids (ggggs) were considered between five antigenic peptides as a linker

Based on in silico analysis, B cell epitopes were predicted in pMGA and PvpA proteins. From pMGA protein with 652aa length, three antigenic regions: amino acids 144 to 274, 394 to 420, and 454 to 480 were selected (Fig. 1A). Two antigenic regions: amino acids 13 to 45 and 62 to 104 were considered in PvpA protein by using the immune epitope database and analysis resource tools (Fig. 1B). Chimeric PvpA-pMGA protein was designed by assembling of five antigenic regions of pMGA_1.2_ and PvpA proteins (Fig. 2).

### Expression of chimeric PvpA-pMGA protein

**Fig. 3:**
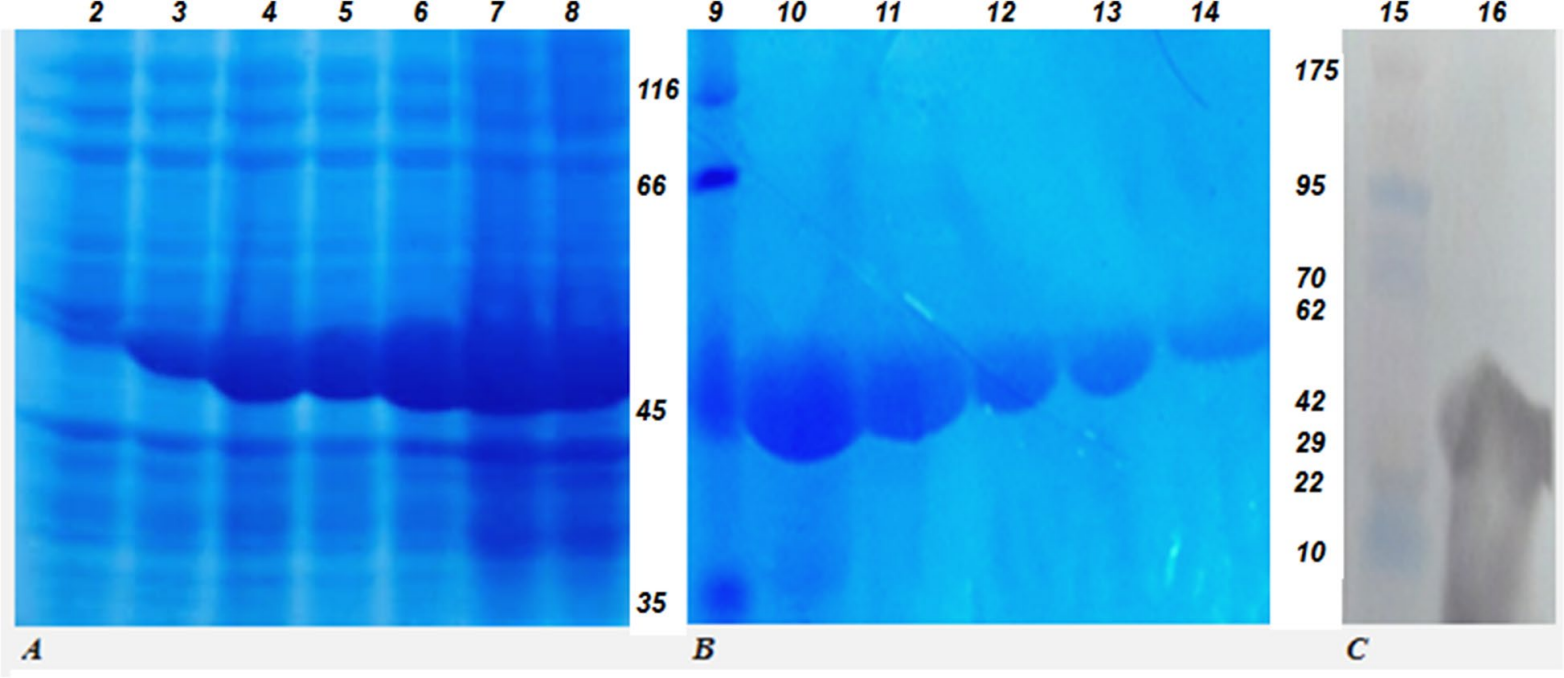
Expression, purification, and western blotting of chimeric PvpA-pMGA protein. **A** Electrophoresis of cell lysate of chimeric protein in SDS-PAGE 10% which was induced in different times; Lane 2: cell-lysated from before induction, lanes 3 to 8 are cell-lysated 1 to 16 h after induction. **B** Electrophoresis of purified recombinant protein. 9: Unstained protein MW marker (Thermo Scientific), lanes 10 to 14 are elutions 1 to 5. **C** Western blotting of recombinant protein by HRP conjugated anti-His tag antibody in nitrocellulose membrane. Lane 15: prestained protein ladder. Lane 16: purified PvpA-pMGA detected by anti-His-tag antibody

The recombinant PvpA-pMGA chimeric protein was induced by 0.1 mM IPTG at 37°C for 16 hours (Fig. 3A). Its apparent molecular mass was estimated to be 47kDa compared to the molecular weight marker (Fig. 3). Analysis of the cell lysate post-sonication demonstrated that the recombinant protein was mostly expressed as an inclusion body. PvpA-pMGA chimeric Protein was purified in the presence of urea (Fig. 3B). The overexpression protein band was observed 1 h after induction. The highest amount of recombinant protein was identified 16 h after induction (Fig. 3A).

### Affinity purification of chimeric protein and immunoblotting

The recombinant PvpA-pMGA protein was purified in the presence of urea 7M by affinity batch formation method (Fig. 3B). The expression of 47kDa recombinant protein was confirmed by using the monoclonal anti-His-Tag antibody in immune blotting (Fig. 3C). High amounts of recombinant protein were observed in five eluted protein from 200μl resin (Fig. 3B). The yield of purified recombinant protein was estimated to be 138 mg per liter.

### Detection of antibody and statistical analysis

**Fig. 4:**
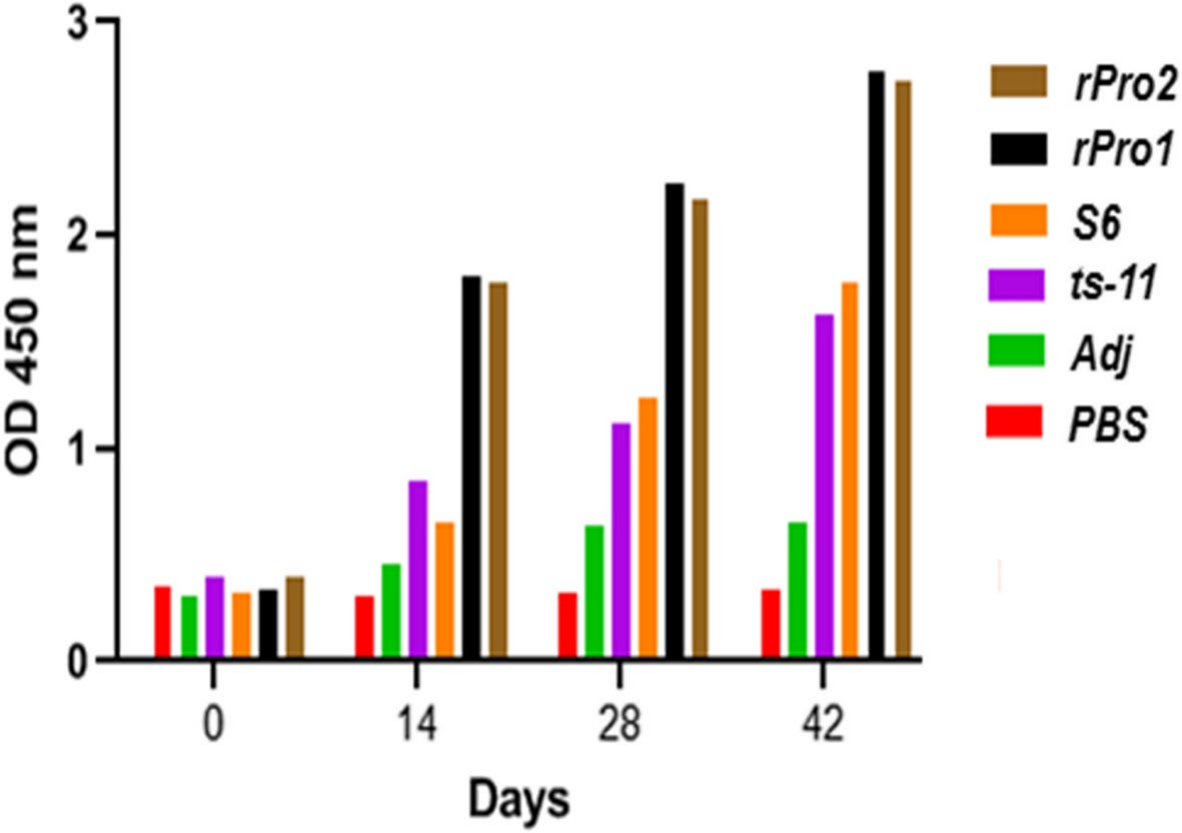
Dynamic changes in the serum antibody levels of immunized chickens. The serum antibody levels were measured by indirect ELISA. The serum was collected at four time points. Day 0, for determination of the preimmunization titer. Numbers represent the mean optical density at 450 nm (OD 450) of serum samples collected at 0, 14, 28, and 42 days after immunization in each group. Chickens were immunized with rPvpA-pMGA (50μg and 20μg), ts-11 vaccine strain, S6 strain, adjuvant, and PBS groups. rPvpA-pMGA (rPro1): 50μg, rPvpA-pMGA (rPro2): 20μg

**Fig. 5:**
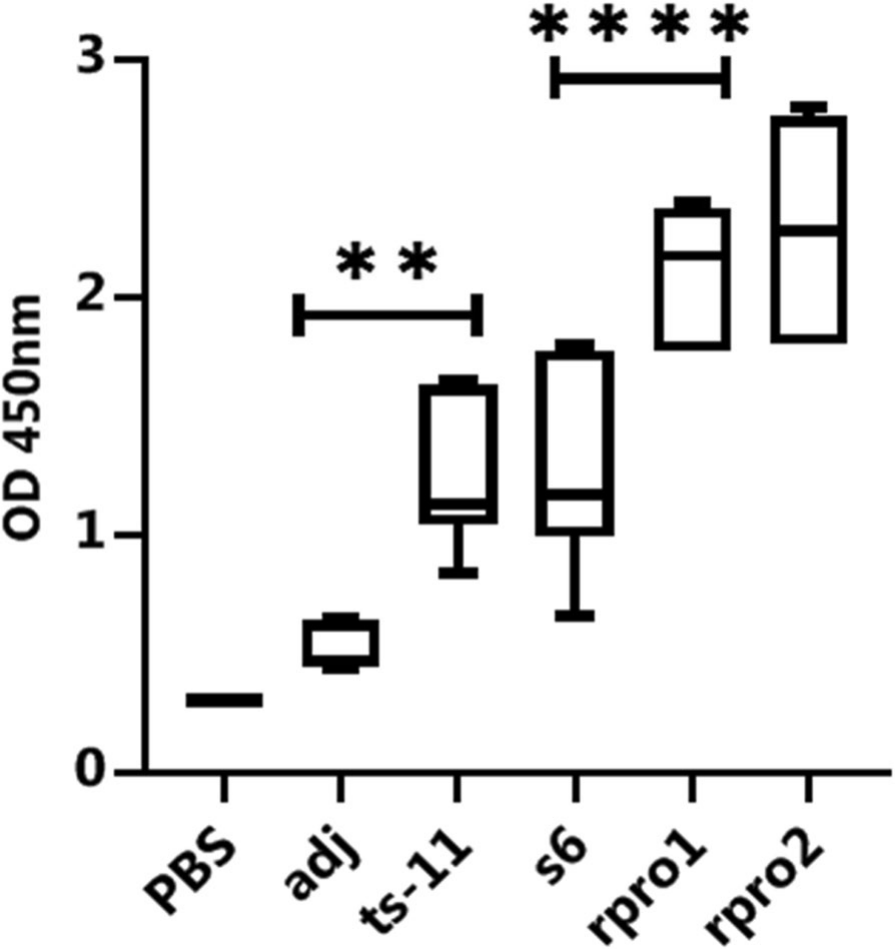
Analysis of IgG antibodies induced by immunization determined by ELISA with recombinant protein in chicken. Numbers represent the mean optical density at 450 nm (OD 450) of serum samples collected at 42 days after immunization in each group. Statistical significance was determined by one-way ANOVA. ^****^P< 0.0001 indicated significant differences

All of the vaccinated animals remained healthy after full vaccination. To investigate the humoral immunity against recombinant PvpA-pMGA protein, the levels of specific antibodies in chicken sera were detected by indirect ELISA on the 14, 28, and 42 days after the first immunization and as shown in Fig. 4, serum antibody levels of the rPvpA-pMGA group, ts-11 vaccine strain, and S6 strain groups exhibited an increasing trend after immunization and were significantly higher than those of the PBS control group. The serum antibody in recombinant protein, ts-11 vaccine strain, and S6 strain groups reached a higher level after booster immunization until 42 days (Fig. 4). The specific antibody titer against PvpA-pMGA recombinant protein in animals that received the *M. gallisepticum* ts-11 vaccine strain and *M. gallisepticum* S6 strain has significantly difference (*p*<0.05, Fig. 5). As depicted in Table 1, there was a significant difference between the means of the antibodies in serum samples of *M. gallisepticum* ts-11 vaccine strain, *M. gallisepticum* S6 strain, rPvpA-pMGA protein, and PBS control groups at 42 days after immunization (*p*<0.05). These results indicated that antigen-specific response was successfully elicited by the rPvpA-pMGA via subcutaneous injection route in chicken.

**Table 1:** Sidak's multiple Comparisons test for six groups based on antibody levels in sera at 42 days after immunization. *p*< 0.0001(^****^), 0.0002(***), 0.0021(**) indicated significant differences

**Sidak's multiple comparisons test**	**Mean Diff.**	**95% CI of Diff.**	**Significant?**	**Summary**	**Adjusted p Value**		
Adj vs. PBS	0.2667	-0.2617 to 0.7950	No	ns	0.725778	B-A	
Ts-11 vs. adj	0.6783	0.15 to 1.207	Yes	**	0.005795	C-B	
S6 vs.adj	0.7083	0.18 to1.237	Yes	**	0.003686	D-B	
rPro1 vs. adj	1.540	1.012 to 2.068	Yes	****	<0.000001	E-B	
rPro2 vs. adj	1.715	1.187 to 2.243	Yes	****	<0.000001	F-B	
rPro2 vs. Ts-11	1.037	0.5083 to 1.565	Yes	****	0.000022	F-C	
rPro2 vs. s6	1.007	0.4783 to 1.535	Yes	****	0.000036	F-D	
rPro2 vs. rPro1	0.1750	-0.3533 to 0.7033	No	ns	0.963656	F-E	
**Test details**	**Mean 1**	**Mean 2**	**Mean diff.**	**SE of diff.**	**n1**	**n2**	**t**
Adj vs. PBS	0.5700	0.3033	0.2667	0.1802	6	6	1.480
Ts-11 vs. adj	1.248	0.5700	0.6783	0.1802	6	6	3.764
S6 vs.adj	1.278	0.5700	0.7083	0.1802	6	6	3.931
rPro1 vs. adj	2.110	0.5700	1.540	0.1802	6	6	8.546
rPro2 vs. adj	2..285	0.5700	1.715	0.1802	6	6	9.518
rPro2 vs. Ts-11	2.285	1.248	1.037	0.1802	6	6	5.753
rPro2 vs. s6	2.285	1.278	1.007	0.1802	6	6	5.587
rPro2 vs. rPro1	2.285	2.11	0.1750	0.1802	6	6	0.9712

Diagnostic sensitivity was estimated using sera collected from the ts-11 vaccine strain and S6 strain groups injected chickens and diagnostic specificity was estimated using sera from preimmunized chickens. The specificity of PvpA-pMGA-ELISA was 100% and diagnostic sensitivity was 100%. The area under the curve (AUC) was 1.000.

## Discussion

*Mycoplasma gallisepticum* contamination in poultry worldwide is a major problem that can result in loss of production and the downgrading of carcasses [57]. The research work is important due to the infections associated with *Mycoplasma* in animals particularly in poultry. The *Mycoplasma* infections can pose a huge economic burden on the farmers and food industry. Increased carcass and downgrading condemnation caused by aereosacculitis, reduced growth and egg production, and induced medication costs make *Mycoplasma gallisepticum* one of the costliest infection illnesses [46, 57].

Comprehension of the risk factors and subsequent decrease of *Mycoplasma gallisepticum* transmission may diminish the risk of contamination. Therefore, it is necessary a powerful method for the detection of *Mycoplasma gallisepticum* in poultry. Since serological monitoring of poultry is utilized extensively as a first-line diagnostic test, there will be an increasing need for characterized antigens to provide the development of efficient and cost-effective control measures [7]. Serological tests including rapid serum agglutination (RSA), hemagglutination inhibition (HI), and ELISA are most generally utilized for monitoring *Mycoplasma gallisepticum* infections [57]. ELISA is a serological test totally specific and sensitive. It was expanded from whole cells and immunogenic proteins from the *Mycoplasma gallisepticum* membrane, as the protein p64 [2, 14]. The following research was designed chimeric protein to develop a recombinant antigen applicable for indirect ELISA to prepare a rapid, accurate, time-saving, and cheap kit for diagnosis of avian mycoplasmosis. The creation of improved serodiagnostic assays that offer optimal specificity in the detection of the highly cross-reactive avian *Mycoplasma* species envisaged with the advent of recombinant DNA technology [5, 42]. Recombinant technology could significantly help to reduce defects, allowing the generation of unlimited amounts of multiple specific antigens [6]. The identification of novel immunogenic proteins is important for the development of both improved diagnostic assays and subunit vaccines. Improvement of recombinant vaccines by cloning and identification of main *Mycoplasma gallisepticum* surface antigens, development of expression, and transformation strategies fascinate the interest of scientists [50, 60, 61]. Mycoplasmal lipoproteins are pro-inflammatory eliciting both innate and adaptive immune responses. Furthermore, the lipoproteins are among the most dominant immunogens in mycoplasmas which have a role in virulence-associated factors including colonization, invasion, and evasion of host defense [47]. Among them, the most abundant is the pMGA or vlhA gene family consisting of 51 genes [52], which encode immunodominant lipoproteins and hemagglutinins [4, 20]. The pMGA genes help the establishment of chronic infection through immune evasion and thus are thought to be significant pathogenicity factors. Researchers have focused on immunogenic proteins of *Mycoplasma gallisepticum*, particularly those engaged with hemagglutination, hemadsorption, and cytoadhesion [38]. Among specific antigens, researchers have identified pMGA (cell surface-exposed lipoprotein), involved in hemagglutination and also pvpA, a putative variable adhesion [10, 36]. According to previous reports, the wild-type GapA, colonization factors, and multiple genes encoding surface antigens have been characterized and cloned [10, 27, 34, 44]. The roles of PvpA, pMGA, MGC2, GapA, and CrmA proteins in the cytoadherence and virulence of *Mycoplasma gallisepticum* have been indicated [15, 25, 37, 58]. The molecular attributes of several surface proteins of *Mycoplasma gallisepticum* including PvpA have been reported [7, 10, 18]. In a previous study, surface proteins P67 (known as pMGA) and P52 were quantitatively purified from the membrane of *Mycoplasma gallisepticum* S6 by a straightforward and non-denaturing chromatographic method [26]. Czifra et al. showed that immunoaffinity-purified pMGA_1.2_ of *Mycoplasma gallisepticum* strain 1226 was able to haemagglutinate chicken erythrocytes [14]. In Buyuktanir et al. study (2008), an enzymatic rapid immune filtration assay prototype was developed to screen *Mycoplasma gallisepticum*-infected chickens by using the purified recombinant PvpA protein [13]. PvpA is a non-lipid integral membrane protein and a putative adhesion in which its species-specific and immunogenic properties have been demonstrated in native and recombinant forms [10, 58]. The pMGA gene family is a large group of surface proteins and is involved in the evasion of the host immune system [5, 20, 52]. Mardassi et al. in 2008 demonstrated the specificity of the *Mycoplasma gallisepticum* hemagglutinin protein encoded by pMGA_1.2_ (a member of the pMGA gene family), and they combined three species-specific purified recombinant proteins (pMGA_1.2_, MS2/28, Mm14) in an ELISA assay to develop rELISA for the simultaneous and specific detection of antibodies to the three major avian *Mycoplasma* species (*M. gallisepticum, M. synoviae, and M. meleagridis*) [6]. Nine members of the pMGA gene family in *Mycoplasma gallisepticum* S6 strain have been sequenced (pMGA_1.1_-pMGA_1.9_), and a high level of sequence identity (>95%) has been found between pMGA_1.1_ and pMGA_1.2_ genes, while other pMGA genes exhibit much lower levels of sequence identity, although there are stretches of amino acid sequence conserved indifferent pMGA surface proteins [37]. It has been indicated that mycoplasmas mostly undergo antigenic variability [15, 41], a mechanism that may affect the sensitivity of serodiagnosis tests that are based on single antigens.

In this study, the novel chimeric protein including three antigenic regions of pMGA_1.2_ and two antigenic locales of PvpA proteins were designed for the evaluation of antibody level against *Mycoplasma gallisepticum* in poultry by indirect ELISA. This led to the conclusion that the combination of two antigens might lead to a more effective detection strategy. The fusion of PvpA-pMGA_1.2_ was constructed for the first time in our survey, according to our knowledge. The availability of the full genome sequence of *Mycoplasma gallisepticum* allows the usage of an immune proteome-based strategy to recognize potential antigenic sites, beneficial to major improvement of diagnostic tests. PvpA protein was found as an unstable protein, and then to resolve this issue, in this study, PvpA-pMGA_1.2_ a chimeric protein based on linear B cell epitopes of two PvpA and pMGA proteins was designed as a stable protein based on analysis by ExPaSy Protein param tool. In addition, the recombinant PvpA-pMGA_1.2_ protein was expressed successfully in *E. coli*. A high amount of chimeric protein was purified at 138 mg/L by affinity method. Although identification of immunogenic antigens and genetic manipulations of *Mycoplasma* in general is more difficult than any other prokaryotic genome, it is vital to successfully express *Mycoplasma gallisepticum* proteins in heterologous systems such as *E. coli* [44].

Antibody response is an important factor when seeking protection against avian *Mycoplasma gallisepticum* infection. In this study, we detected the high level of antibodies induced by rPvpA-pMGA protein and found that it had the ability to stimulate humoral immune response following immunization; the rPvpA-pMGA successfully elicited the IgG(Y) response via subcutaneous route immunization in chicken. The results showed that compared to the PBS control group, the serum antibody levels of the recombinant protein, ts-11 vaccine strain, and *M. gallisepticum* S6 strain groups were significantly increased, within 14 days from the first immunization (Fig. 4). This was found to be in agreement with El-Shater et al. [16] who mentioned that vaccination of chickens with *M. gallisepticum* subunit vaccine resulted in high antibody response at 2 weeks after the booster dose. The serum antibody in both of recombinant protein group and vaccine group reached a higher level after booster immunization until 42 days (Fig. 4). As depicted in Table 1, there was a significant difference between the means of the antibodies in serum samples of chimeric rPvpA-pMGA protein *with another* ts-11 vaccine strain*,* S6 strain, and PBS control groups at *p*<0.05. These results indicated that antigen-specific response was successfully elicited by the rPvpA-pMGA via subcutaneous injection route in chicken. This study has shown, for the first time, that a degree of immunity in chickens can be produced by immunization with chimeric PvpA-pMGA protein. The recombinant protein PvpA-pMGA used in immunization offered an increasing trend significantly higher than those of the ts-11 vaccine and S6 strains group as shown in Fig. 4. These data indicate that the rPvpA-pMGA protein can induce positive immune responses, resulting in a significantly increased antibody level. Immunoinformatics tools allow the detection of pathogens underlying immunogenic proteins, together with the prediction of diverse immune-dominant epitopes which are involved in the development of humoral and cellular immune responses against the pathogen. For that reason, a multi-epitope-based peptide vaccine can be designed with immunogenic proteins of a pathogen. Very few studies are reported in the field of in silico vaccines for poultry and animals. The studies validate the immunoinformatics method to design multi-epitopic vaccines against infectious diseases in poultry [43, 53], and research on in silico approaches had not yet been reported for *Mycoplasma gallisepticum*, while some researchers used in silico approaches for the identification of virulence candidates for other *Mycoplasma* species such as *Mycoplasma pneumonia* type 2a strain 309 and *Mycoplasma agalactiae* [19, 49]. Thus, studies on in silico approaches are needed for the development of effective vaccines. The in silico validations were included in this study.

The currently available live attenuated vaccine and bacterins are commonly utilized in commercial birds; these vaccines cannot aid in control during the sudden onset of *Mycoplasma gallisepticum* infection; strict biosecurity has to be followed to control and eradicate the infection. The live attenuated vaccine shows adverse side effects and pathogenicity, while the bacterins are associated with the high cost and repeated dosage. Hence, new novel recombinant vaccines are required to be developed which are more efficacious and less expensive. The research conducted will add value to the existing pool of scientific knowledge. In this research, diagnostic sensitivity and specificity were estimated using sera collected from ts-11 vaccine strain and S6 strain groups injected chickens as a positive and PBS group sera used as a negative. The specificity and sensitivity of PvpA-pMGA-ELISA were 100%. Also, Wanasawaeng et al. [54] indicated that the ELISA prepared with field strain revealed high sensitivity and specificity compared with the commercial ELISA test (67% and 95%, respectively). The area under the curve (AUC) obtained in this study is considered good (1.000) having the ability to classify and thus avoid a false classification, taking into account the positive and negative samples [59]. This study showed that chimeric PvpA-pMGA protein can be used to detect specific antibody levels in vaccinated chickens with tS-11 and S6 strains. Further study is needed to evaluate the possibility of diagnosing infected flocks by PvpA-pMGA-ELISA. This novel chimeric protein could be useful in indirect ELISA tests for the evaluation of antibody levels in vaccinated poultry flocks.

## Conclusion

This study shows that the pET32a-PvpA-pMGA recombinant clone was successfully expressed and stable in *E. coli* DE3 host cells. The chimeric PvpA-pMGA protein can be used to detect specific antibody levels in vaccinated chickens with tS-11 and S6 strains. Further study is needed to evaluate the possibility of diagnosing infected flocks by PvpA-pMGA-ELISA. This novel chimeric protein could be applicable in ELISA for the evaluation of antibody levels in vaccinated poultry flocks.

## Data Availability

Not applicable.
